# Analysing the landscape for prescription to non-prescription reclassification (switch) in Germany: an interview study of committee members and stakeholders

**DOI:** 10.1186/s12913-019-4219-6

**Published:** 2019-06-20

**Authors:** Natalie J. Gauld

**Affiliations:** School of Pharmacy, The University of Auckland, Park Rd, Grafton, Auckland, New Zealand

**Keywords:** Nonprescription drugs, Self medication, Access to medicines, Health policy, Medicines regulation, Reclassification

## Abstract

**Background:**

Non-prescription medicines are increasingly used in Germany, aided by prescription-to-non-prescription reclassification (or switch). This study aimed to examine the barriers and enablers to reclassification of medicines in Germany and provide recommendations for change.

**Methods:**

Face-to-face conversational interviews with purposively selected key informants in Germany were conducted in 2017 by a researcher informed in the area. Interviews were transcribed, coded in NVIVO and systematically analysed using a framework approach.

**Results:**

Twenty-four interviews were conducted with 32 participants including members of the committee considering reclassifications, and representatives from government, industry, health insurance, academia, and pharmacy, medical, and patients’ organisations.

A range of enablers and barriers emerged that influence reclassification including effects on the committee and process, or the desire of pharmaceutical companies to pursue reclassifications. Enabling market factors included the large population and a culture of self-medication. Enabling health system factors include the pharmacy-only category. Some pharmacy factors appeared enabling (e.g. a positive experience after reclassifying emergency contraception) while others appeared to hinder reclassification (e.g. insufficient pharmacy practice research). Some medical factors were enabling (e.g. reported waiting times) and others limited reclassification (e.g. opposition to some reclassifications). Some committee and government openness to reclassification and self-medication reportedly enabled reclassification, while conservatism was considered a barrier, particularly for classifications with special conditions for supply such as initial doctor diagnosis or other complexities. Some improvements to the committee constitution and considerations were recommended. Some participants found the reclassification process after the committee recommendation opaque, with opportunity for delays and political interference.

Industry factors included both enablers such as capability in reclassification, and barriers, such as a perceived low market potential of some reclassifications, and doubt that some candidates would be approved.

A need for more data emerged strongly, both pre-reclassification in applications, and post-reclassification.

Many participants saw merit with reclassification in non-traditional areas such as hypertension, diabetes and oral contraception.

**Conclusions:**

Many factors influence reclassification in Germany. Recommended improvements included aspects of the process and committee consideration, and more data collection. Sufficient market exclusivity linked to data collection could aid the generation of evidence to aid committee considerations and encourage more applications of high quality.

## Background

People commonly manage illnesses with self-care [1]. Such care includes self-medication, which the World Health Organisation defines as the use of approved medicines that are available without prescription to treat their ailments and conditions [2]. For example, in Germany 46% of adults use non-prescription medicines in a week, similar to prescription medicines [3]. Reclassifying (or switching) medicines from prescription to non-prescription is a likely contributor to the ability to self-medicate [3], and part of an international trend [4].

Potential benefits of reclassification include timely and convenient consumer access to medicines, public health benefits (e.g. increased smoking cessation) [5], and savings for health funders [6, 7]. A recent sildenafil reclassification in New Zealand was associated with reduced internet purchases [8], and referral of men to the doctor, for example for high blood pressure [9]. Risks include inappropriate use, delayed diagnosis, misuse and adverse events [6, 7].

Although countries consider similar criteria in assessing reclassifications [10, 11], reclassification activity varies between them [12]. Germany has been advanced in reclassification [13], but was 10–12 years behind Australia, and the United Kingdom (UK) for emergency contraceptives, despite early reclassification attempts in Germany [14–16]. Although having switched fewer medicines [13], France reclassified the emergency contraceptive pill in 1999 [16], 14 years before Germany. Germany also rejected reclassifying dermal adapalene (for acne) in the same year as the United States (US) and New Zealand approved the reclassification [17].

Analysis of meeting minutes in Germany found some departures from the European Union (EU) switch guideline, that the committee considering reclassifications commonly requests more data, and that their recommendations are not always followed [15, 18]. Record analysis in the US similarly found a departure from the key questions and principles for reclassification by the committee, variability, and a lack of uniformity and transparency [19]. Stakeholder interviews suggest reasons for the variation in reclassification between countries include health system factors, pharmacy-only schedules, population size, cultural aspects, committee factors, government interest, medical and pharmacy influence, and confidence in consumers and pharmacy [12, 20]. A survey of US Food and Drug Administration (FDA) advisory committee members (limited by a 5% response rate) found some deficiencies in preparing for meetings, and strong influence of the FDA documents and presentation [21]. In Germany, despite being the fourth largest pharmaceutical market in the world, the influences on the committee, or on reclassification generally, have not been investigated.

In Germany, anyone can submit a switch proposal. Bundesinstitut für Arzneimittel und Medizinprodukte (BfArM), one of the medicines regulators, evaluates it, producing a report and recommendation [22, 23]. A 23-member expert advisory committee, chaired by a BfArM employee, considers human and veterinarian medicine reclassifications. Voting members comprise academic members representing pharmacology, clinical pharmacology, veterinarian pharmacology, pharmacy, clinical pharmacy, internal medicine, general medicine, paediatric medicine, medical statistics or epidemiology, and veterinary medicine, and nominees of the Drug Commissions of Physicians, Pharmacists and Veterinarians. Non-voting members comprise a general practitioner, an internal medicine specialist, paediatrician, pharmacist, a veterinarian, a dentist, a naturopath, and representatives of the human (*n* = 2) and veterinarian (*n* = 1) pharmaceutical industry. The committee consideration uses EU criteria [24] then makes a recommendation, by majority vote. The Ministry of Health then progresses a change if they choose to, preparing a draft ordinance and consulting various stakeholders, then seeking the consent of the Federal Council (Bundesrat).

Germany has three categories of medicines: prescription medicines (Verschreibungspflichtige Arzneimittel), pharmacy-only medicines (Apothekenflichtige Arzneimittel) and general sales medicines (Freiverkäufliche Arzneimittel). Most non-prescription medicines are only available in pharmacies. Pharmacy-only medicines can be supplied in a pharmacy by a pharmacy assistant or pharmacist. Medical visits are free to patients, and patients pay around €5–10 per funded prescription item, depending on the cost of the item. Non-prescription medicines are typically not funded on prescription, except in children up to 12 years old (or 18 years with developmental disorders). There is no funded minor ailments service in pharmacies.

This research aimed to explore the barriers and enablers to reclassification of medicines from prescription to non-prescription in Germany from the view of stakeholders and provide recommendations for improvements.

## Methods

Following informed consent, face-to-face interviews were conducted in late 2017 with people particularly informed about reclassification in Germany, or who represented stakeholders.

Participants were purposively selected with input from the scientific advisor in a maximum variation sample [25] to capture multiple perspectives including: committee, government, pharmacy, industry, academia, consumer, health insurance and medical. To include those most informed about the topic [26], most participants were from the first four groups. Committee members were selected from the list of committee members and alternates published by BfArM, “Die Sachverständigen-Ausschusses für Verschreibungspflicht”, excluding veterinary experts, naturopaths and dentists. Pharmacy, consumer, patient, and medical organisations were contacted with representatives invited to participate. A health economist, a leading pharmacy academic, a health insurance spokesperson, and a politician were also approached.

The research used an ‘insider’ approach [27] with an interviewer experienced in the topic, who had been a classification committee member, had reclassified medicines, and conducted similar research elsewhere. This ensured time-efficiency for participants through understanding concepts discussed, and aided the conversational style interview. Interpretive description [28] was used to consider the application of the research in participant selection, interview topics, analysis and reporting.

Topics explored are provided in Table 1. The interview content varied with the participant’s role to maximise learning, follow the natural conversation direction, and explore themes emerging in previous interviews. Notes were taken for one interview, the remainder were recorded with permission and transcribed verbatim. Participants were given their transcripts to review, and were sometimes asked further questions or for clarification. Some quotes and sections were confirmed with some participants during reporting on participant request and/or for fact-checking.

**Table 1:** Topics typically covered in the interviews

• Barriers and enablers to reclassification in Germany	
• Experiences of the processes and committee meetings	
• Potential improvements to the process or constitution of the committee	
• Quality of applications	
• Views on reclassification and self-medication generally	
• The ability of pharmacy to manage non-prescription medicines	
• Access to doctors in Germany	
• Consumer culture and behaviour	
• Research on reclassifications	
• Market exclusivity related to collection of evidence	
• Potential areas for reclassification.	

Transcripts and notes (for one interview) were coded using Nvivo 11 and analysed thematically using the Framework approach [29], with themes derived deductively or from related work from the researcher [12]. Themes were worked through systematically, with constant comparison within and between the different groups of participants. Commonalities were explored, as well as exceptions and contrary cases. No document analysis was undertaken.

An overarching figure was derived from the enablers and barriers most often arising or appearing most salient throughout the analysis. Feedback on this figure was sought from participants who were particularly well-informed in reclassification (representing regulatory, industry and pharmacy expertise) before it was finalised.

Professor Broich, President of BfArM, was scientific advisor, and reviewed the planned list of participants, question guide, and draft report before each was finalised.

## Results

Most interviews (Table 2) took one hour (range 20 min to 2.5 h). Some participants invited others to their interview, in a secondary role. All took place in English except one in German with an interpreter.

**Table 2:** Participants

	Number of interviews	Number of participants
Committee members	10	10
Consumer or patient organisations	1	1
Pharmacy Academic who was not a committee member	1	1
Industry – not on committee	3	5
Pharmacy – not on committee	3	5
Medical organisation representatives – not on committee	1	2
BfArM or Ministry of Health staff member	3	5
Health Insurance representative	1	1
Health Economists	1	2
Totals	24	32

*BfArM* Bundesinstitut für Arzneimittel und Medizinprodukte

This paper outlines influences on reclassification activity in Germany. It focuses on the three key areas arising in interviews: the process; the health system; and influences on reclassifying in companies. It also includes key recommendations from the participants.

### Factors influencing reclassification activity

**Fig. 1:**
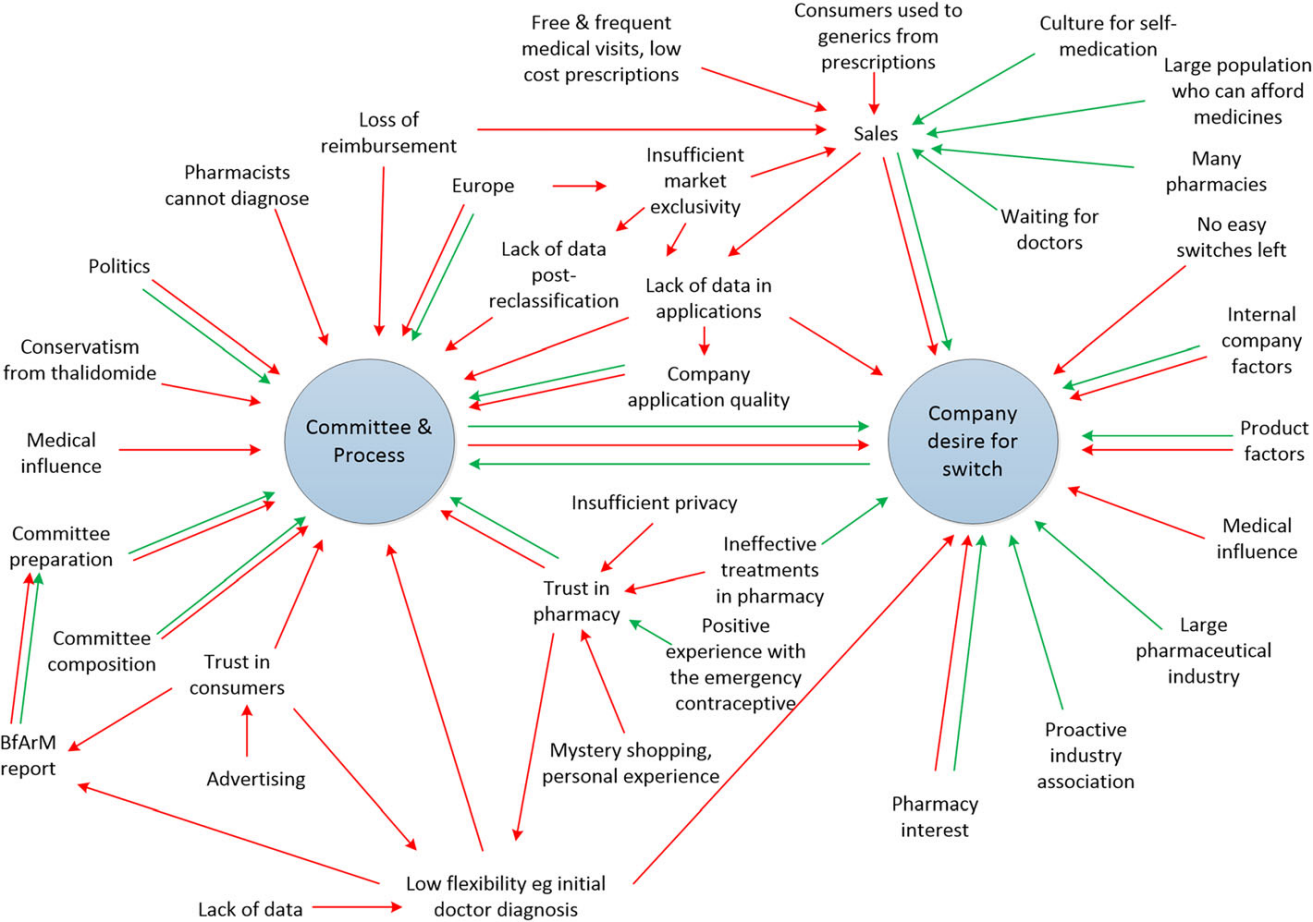
Overview of many of the barriers (red) and enablers (green) for reclassification in Germany

A complex array of many enablers and barriers arose from the interviews (Fig. 1). Enablers are presented in green and barriers in red. The strongest, observed by most participants, was a need or desire for more data pre-reclassification and/or post-reclassification. Commonly observed influences on reclassification included a high frequency of doctors’ visits, aspects of the health insurance model, pharmacy aspects and politics, with influences often hindering it, and sometimes encouraging it. The large self-medication market, and aspects of the process e.g. scientific advisory meetings and having an expert attend the meeting emerged as important enablers. These have been discussed below within the framework outlined in Table 3.

**Table 3:** Framework for describing enablers and barriers to reclassification emerging from the interviews

• Process and Committee	
◦ Scientific advisory meeting	
◦ Application	
◦ Need for evidence	
◦ BfArM evaluation	
◦ Committee membership	
◦ Committee meeting	
◦ Committee trust in consumers, pharmacists and companies	
◦ Conditions or guidelines for supply	
◦ Attendance at the meeting by an expert for the company	
◦ Post-committee process	
• The Health System including Funding	
◦ Health funding	
◦ Access to health care	
◦ Medical influence and perspectives	
◦ Pharmacy influence and perspectives	
• Industry aspects	
• Other factors	
◦ Consumer culture and behaviour	
◦ Politics	

### The process and committee consideration

Many aspects about the process and the committee consideration arose in the interviews as enabling or hindering reclassification.

#### Scientific advisory meetings

Participants reported that BfArM had recently encouraged scientific advisory meetings allowing pharmaceutical companies to meet them regarding potential reclassifications. Government participants considered that these meetings aided the application quality and helped companies decide whether to pursue a reclassification. Industry participants appreciated receiving input on research and other aspects, and discussing the reclassification possibility with the regulator.

*…if there are questions around a switch, how to prepare, which areas to evaluate… then it would be helpful for the companies to ask for scientific advice first…. there might also be situations where the company then understands … why a switch might be quite difficult and … it is not worth to submit an application… (Government participant)*

#### Applications

Committee members were equally divided between being satisfied with the company applications, and those considering at least some needed improvement*.* Justification for the reclassification and more data were the key themes for the latter group, although even some who were satisfied with the applications wanted more data. Data and evidence are discussed further below. Some government participants reported variability in applications, with some quite good, but others quite poor. A quality application with good data, strong justification for the reclassification, and a relatively straight-forward reclassification in an area typically managed by self-medication all reportedly helped gain approval.

#### Need for evidence

Evidence was discussed frequently regarding the application and committee consideration. It also arose regarding post-reclassification usage and safety and evidence for products supplied in pharmacy.

Good evidence reportedly helped the committee recommend approvals for emergency contraceptive and nasal corticosteroids reclassifications. Some participants noted the need for the committee to be evidence-based (and reported that it sometimes was not), and there were suggestions of committee selectivity in what evidence they used. For example, some participants noted that evidence of harms from other countries was accepted, but positive evidence from elsewhere or observational data was less accepted. Evidence from countries deemed to be unlike Germany was disregarded, and one committee participant suggested even Switzerland or Austria could differ from Germany.

Most participants wanted more data. While one industry participant considered their applications had sufficient data, other industry participants indicated more data would help some applications, but incentives were needed given the costs, and immediate generic competitor entry upon reclassification limiting the market potential. A committee member stated that insufficient data increases use of personal opinion on the committee and affects objectivity. Participants from the committee, health insurance, government and a patient organisation wanted post-reclassification research given insufficient German pharmacovigilance data and “*…we don’t know how these people take these drugs”*. Several participants considered such research could aid later committee reviews of a reclassification, particularly with difficult decisions, but one participant worried it could enable reclassifications with insufficient safety evidence. Another said BfArM would advise the committee of concerns, making such research unnecessary.

Although more data was desired pre- and post-reclassification, barriers to pharmacy-based research emerged, e.g. being less attractive to academics than pharmaceutical science research, and low pharmacy participation. Observational research was suggested to be neither common nor particularly trusted in Germany generally. An industry participant suggested conducting pre-reclassification pilot studies, but noted they were currently impossible.

#### BfArM evaluation and recommendation

The committee members valued BfArM’s evaluation and recommendation, although one voting committee member reported occasional evaluation deficiencies. Industry participants wanted the evaluation report shared with the applicant in advance to be better informed before their nominated person presented to the committee.

*…[it] is hard to present something in the committee when they do not know what’s in the assessment report. What are the obstacles… that the authority sees… Sometimes you are completely wrong [about] where the problems are. (Industry participant)*

However, government participants had reservations, that new information would then be presented at the meeting, or that BfArM could appear to have already negotiated with the applicant, undermining the committee’s work. Another participant suggested that advance provision of the evaluation might result in companies approaching committee members. While some voting committee members saw no need for advance provision to companies, e.g. because the company could just apply again, two saw benefits:

*… there are quite long terms between the meetings and I think the company would prefer to get some more information as early as possible, so I would support this… In most cases we have some questions and the company cannot answer the questions in this moment…*

#### Committee membership

Most participants commented favourably on committee membership, e.g. the range of areas represented. However, one non-voting member reported feeling *“not a real member”*, and some non-voting members and their alternates reportedly missed some or even most committee meetings. Allowing non-voting health practitioners to vote was suggested to aid engagement, although several participants acknowledged potential conflicts of interest.

*And in a certain way you cannot get rid of these conflicts of interest as you need people who know the medical situation and… have … practical experience, … office-based physicians then of course they look for their economic interests too. (Voting committee member)*

Some committee participants (but not all) supported splitting the veterinary functions from the human medicine functions, some quite strongly. This was motivated by the large size of the committee, expertise, or reported vested interests from veterinarians to reject some reclassifications.

*…we have to listen to some drugs for snakes or some drugs for fish and… I have no idea what to vote for. And if I look at those [veterinarians] voting on human medicines, I think it’s very similar. (Voting committee member)*

Committee members wanted to hear the patient or consumer voice, but thought the scientific discussions were too difficult for such a representative. A health insurance participant noted the consumer's increasing importance, and inclusion on other medicinal committees. Conversely, the patient participant worried about finding an appropriate person for the range of medical conditions, and their organisation had other priorities. This participant also noted that patient groups were consulted after the committee meeting, as acknowledged by a government participant, thus representation on the committee was deemed a low priority for their patient organisation.

Some participants wanted a more practical than academic focus for committee members, for example no practising community pharmacist attended the committee. One questioned the need for a hospital pharmacist or a second pharmacologist:

*… the committee consists [of] people [who] do not have ordinary day-to-day practice with patients, especially patients at the borderline of just OTC or just not OTC. (Committee member)*

#### Committee meeting

Most committee members reported that the committee performed well in the meeting, some noting an increasing scientific-basis.

*…we had not one case where we had to revoke one of our votes…* (Committee member)

However, several participants spoke of strong personal beliefs occasionally swaying the committee. Two industry participants doubted the scientific basis of some decisions. An industry participant reported a reclassification was rejected although “…*all the questions could be answered by the documentation”.* The same data resubmitted resulted in an approval.

Various participants recommended the collection of more data (see below), with some noting that it would encourage evidence-based decision-making.

While some committee members were reportedly *“very well prepared”*, some suggested others could be better prepared, with doubts sometimes expressed that the application was read sufficiently. Two participants noted the minimal payment to members (€30).

The committee sometimes reportedly deviated from the EU switch guideline, discussing societal implications (e.g. with the emergency contraceptive), reimbursement concerns and medicine effectiveness. While discussions of the loss of reimbursement post-reclassification and equity of access were reportedly truncated (being outside the guidelines), many committee members were suggested to *“have that in mind”*. Two committee participants reported considering effectiveness, despite being outside the guidelines, feeling it was important. Departing from the guidelines was attributed to insufficient guideline knowledge, and *“our prejudices and … our own ways of assessing risk”*. Some participants wanted a benefit-risk assessment rather than only a risk assessment.

A committee member and an industry participant suggested that there was some committee overreliance on the evaluation report, sometimes to the exclusion of the company application, especially with a full agenda. While votes were usually aligned with the BfArM recommendations, a participant reported:

*There are always surprises here in this committee…. And it depends on… sometimes only one question of the specialist at the table. (Committee member)*

The 2016 rejection of adapalene (a topical retinoid for acne), contrasting with approvals in two other countries, was attributed by several participants to the history with thalidomide. Thalidomide, a teratogen initially available without prescription, had been developed by a German company. One participant suggested this history caused Germany to be more conservative in general than other countries.

#### Committee trust in consumers, pharmacists and companies

Some committee concerns about the consumer, pharmacist and companies negatively influenced decisions. Two committee participants worried that consumers could buy many packs of a medicine from different pharmacies. Others suggested consumers would lie, with one participant suggesting that women would lie about their weight when requesting the emergency contraceptive, potentially causing failure of the medicine. Doubting consumers could self-diagnose hayfever, the committee decided initial doctor diagnosis was required.

*We have to trust the consumer, but we have also to trust the [pharmacist], and … the companies and … the advertising chapter of the company. (Committee member)*

Committee members held mixed views on pharmacist capabilities. One committee member suggested *“the general trust for [the] role of pharmacists in the committee is limited”*, noting evidence of ability with reclassified medicines would help. Some mentioned negative mystery shopping results, personal experience of poor performance, or that non-pharmacist staff were seen more than pharmacists. However, various committee members were positive about the role of pharmacists, considering pharmacists could be used more, or become prepared for reclassifications with further training. Two committee members and a government participant observed that pharmacists did their jobs as well as other health professionals, or that some doctors also had deficiencies. As pharmacists could not diagnose by law, there was a general expectation that *“the product [needed to be] suitable for self-diagnosis”* rather than using pharmacists to diagnose.

#### Conditions or guidelines for supply

Some reclassifications were enabled within the committee and later process by requiring initial doctor diagnosis, e.g. triptans and nasal glucocorticoids. Industry and some committee members saw further opportunities with this, but government participants considered them exceptional. Some participants indicated a protocol for supply could enable reclassifications, but government and pharmacy participants were largely unenthusiastic:

*…we need to be careful that we don’t provide a number of criteria and conditions under which the drug can be sold on a non-prescription basis because sometimes it’s getting bigger and bigger, so the conditions can then no more be handled. (Government participant)*

Unlike other German reclassifications, the emergency contraceptive had a non-mandatory guideline for pharmacists, including documenting the consultation, and advertising and mail order were not allowed. Pharmacy organisations had provided training. Many participants (but not medical organisation participants) considered pharmacists had managed this reclassification well.

#### Attendance at the committee meeting by an expert for the company

Participants reported that, with committee permission, industry may send an expert (usually an external specialist) to the committee meeting to present for 10 min and answer questions, leaving before the BfArM presentation or discussion. While found useful, some participants reported experts appeared biased, had occasional knowledge deficiencies or could focus their presentation on the wrong areas. An industry participant suggested a company regulatory staff member also in attendance could help answer queries, which several committee members saw merit in.

#### Post-committee process

The Ministry of Health or parliament decision can differ from the committee recommendation. Some participants were concerned or frustrated by the process in terms of complexity, insufficient transparency, unexplained delays, or no reclassification following a positive committee recommendation, and commercial ramifications of delays, sometimes of years.

*…one peculiarity is that law makers do not have to follow our suggestion. (Voting committee member)*

A government participant noted that unlike the committee’s scientific perspective, social, cultural and religious aspects were considered afterwards. Various participants attributed this differing decision or unexplained delays to politics including medical lobbying, a conservative government and religious influence. The calcipotriol (for psoriasis) rejection reportedly followed dermatologist pressure after the committee recommendation to approve the reclassification. The Ministry of Health rejected the *“controversial”* emergency contraceptive reclassification following positive committee recommendations in 2003 and 2014. This caused *“a blockage between the government, the Ministry of Health and the second chamber of Parliament which needs to approve our ordinances.”* However, after the EU agreed to reclassify ulipristal in late 2014, the Ministry of Health supported the reclassification of ulipristal and levonorgestrel, reportedly stating that pharmacy could manage emergency contraception.

While participants accepted the first part of the process involving BfArM, the evaluation, and the committee consideration, change was desired for the post-committee process. A government participant and an industry participant suggested changing to a higher Federal authority decision alone without a committee or the Ministry process, to improve decision timings. Others wanted transparency in outcomes and reasons, and time-lines to be provided.

### The health system including funding

The health system, including health funding, medicine and pharmacy, appeared very influential in whether a reclassification would be sought, and its commercial success.

#### Health funding

Reimbursement for a prescribed medicine is lost with a reclassification, except in children, hindering some reclassifications, particularly for chronic conditions. Companies reportedly decided whether the market opportunity is better under the reimbursed model or reclassified with lost reimbursement. Some recommended that reimbursement was not lost automatically post-reclassification.

Many noted that, despite potential savings, neither the health insurer nor politicians were promoting reclassifications. Two participants suggested that health insurance’s large surplus minimised the need for reclassification. Others suggested that capped health insurers’ payments to doctors limited potential savings from reclassification, and the insurer’s costs could rise, e.g. from misdiagnosis or misuse, or companies promoting newer, more expensive medicines to doctors instead of those reclassified.

*One of the points… is always when the OTC [over-the-counter] drugs are misused and afterwards the statutory health insurance system has to pay… (Health insurance participant)*

Health funding also contributed to use of doctors and prescribed medicine rather than self-medication.

*…we’re quite happy with the situation to have access to the medicines even if you’ve got to go to the doctor to get it. Because it’s better to have it free or cheap… (Patient participant)*

#### Access to health care

Some participants, including the medical participants, perceived a low need for reclassification, noting the good health system, mostly good access to doctors, and/or sufficient non-prescription treatments. Conversely, others observed that delayed doctor access and waiting times, particularly in rural areas, alongside pharmacy accessibility meant reclassification aided consumer convenience. Various participants recommended better access to medicines such as chronically used medicines through reclassification.

*…it’s not so easy to go to get an appointment. (Patient participant)*

#### Medical influence and perspective

Various participants suggested that doctors hindered reclassification, attributing it to loss of income or control, or distrusting consumers. One doctor reported that general practice relies on three-monthly attendance for prescriptions, so a reclassification could reduce income, and others reiterated this view. An alternate view was that, with only one visit funded per quarter per patient, doctors could benefit from fewer unfunded appointments. Although two doctors raised insurer-imposed constraints on prescribing budgets, with frustration, they did not suggest reclassification could help.

The medical organisation representatives reported that their opposition to the emergency contraceptive reclassification was not financially-motivated, noting that five emergency contraceptive supplies per gynaecologist is *“… not much money.”* They noted more accessible gynaecologists in Germany than elsewhere, reducing the need for reclassification. They also reported a culture in Germany to use the gynaecologist for contraception, and stated that abortions had increased post-reclassification. They worried about not knowing what the patient bought from the pharmacy, and considered that many medicines available without prescription should not be, e.g. vaginal antifungals and paracetamol.

Indirect medical influence also occurred. The need to retain doctor relationships reportedly stopped pharmaceutical companies pursuing some reclassifications, e.g. oral contraceptives, antihypertensives and lipid-lowering agents.

#### Pharmacy influence and perspective and the pharmacy-only category

The pharmacy influence was complex. Various participants considered the pharmacy-only category enabling, not wanting *“mass market”* medicines. Mixed confidence in pharmacy affected some participants’ support for reclassifications. Negative influences were mystery shopping results or sales of remedies that were not evidence-based in pharmacy. However, a government participant and others considered pharmacy had performed well with emergency contraceptive supplies, and a participant reported that 90% of women getting emergency contraceptives used a pharmacy without a prescription. Pharmacy organisations had supported pharmacy in providing emergency contraception with trainings and a guideline, reportedly well-received. Some participants suggested the guided pharmacy consultation for emergency contraception was better than with some doctors, e.g. after-hours doctors specialising in other areas. The inability for pharmacists to diagnose was considered very difficult to change given its long history and likely medical resistance to a change. Various participants including committee members considered pharmacists were capable of doing more than what they were currently doing. Some participants recommended further training for such roles, or better undergraduate pharmacy practice education.

Pharmacy participants appeared overall ambivalent about reclassification for various reasons:

*When the product is [prescription-free] you get less… and we have more Verantwortung [responsibility]…*

Pharmacy discounting of non-prescription medicines, and loss of non-prescription sales to mail order/internet pharmacies made dispensing (with less responsibility, liability, and a certain return) more attractive.

One pharmacy participant’s reluctance for a *“third class”* of medicine with screening tools and documentation arose from concern other medicines would move to the mass market. Pharmacy reluctance for reclassification arose from wanting not to alienate doctors or believing sufficient medicines were already available without prescription. In contrast, most pharmacy participants were positive about potential reclassifications they considered useful, e.g. antibiotic eye drops, or a stronger dermal steroid.

### Industry aspects affecting reclassification

Industry applications were recognised as important for driving reclassifications. Industry participants were positive about reclassification, wanting to provide a more effective or convenient product than existing non-prescription medicines.

Company attributes, product attributes, general market factors, health system, feedback from the scientific advisory meeting, and the committee and reclassification process affected companies’ reclassification decisions (Fig. 1). Some companies exclusively focused on prescription opportunities, without interest in or capability with non-prescription medicines or reclassification. The large German market and company enthusiasm for reclassification helped stimulate discussion about reclassification opportunities.

Participants revealed many factors that made potential reclassifications commercially unattractive. These included: immediate competitor entry after reclassification; a product with a small market size in a niche area; the company having no known brand for consumer recognition; possible medical backlash; and loss of health funding mentioned above. An example provided was the omeprazole reclassification which was described as non-viable for the company that initiated it. Cheap immediate non-prescription generic competition, and the consumer advantage of low cost for large amounts prescribed also reportedly inhibited non-prescription sales of that company’s brand. This brand was withdrawn and the company became wary of future reclassifications.

*…you are working for the total industry even if you invest your money and so I guess a lot of companies just wait for activities from others. (Industry participant)*

Company reclassification applications had become fewer, possibly related to industry frustrations from process delays, previous disappointing outcomes, and that the ‘easy’ reclassifications had been done. Despite a recent positive UK decision, no sildenafil reclassification was pursued in Germany, with doubts it would be approved. Industry participants reflected on perceived regulator and committee reluctance to use initial doctor diagnosis as a supply condition. Some felt that this would make reclassification too difficult for less straight-forward reclassifications that might need special conditions for supply, such as initial doctor diagnosis, for example with sildenafil.

Industry found the Europe-wide one-year market exclusivity too short. They preferred three years’ exclusivity, which could reportedly enable pre- and post-reclassification research if available.

### Other factors

Aspects of consumer culture and behaviour and politics have been reported above under the various topics.

A very large self-medication market reportedly arose given the population size, affluence, and self-medication culture in some consumers. Conversely, very frequent doctor visits and using prescribed medicines rather than self-care reportedly reflected free medical visits, low prescription co-payments, a culture to readily consult doctors, consumers wanting benefit from their medical insurance payments, and employers' needs for doctors’ sickness certificates.

Politics arose in various ways, mostly negatively affecting reclassifications, e.g. the emergency contraception reclassification rejection despite a positive committee recommendation. Politics were suggested to arise from boundaries and control between professions, and contribute to pharmacy reticence regarding reclassification.

## Discussion

Reclassification in Germany has many influences. Some aspects of the process and committee consideration were largely well-regarded. The population size was enabling, and influences (aiding and/or hindering reclassification) were described relating to the process, the EU, and medical, pharmacy, consumer, insurance and industry aspects. Improvements were identified for applications (e.g. more data), the process (e.g. transparency and clear timelines), committee composition, and the committee consideration (e.g. review of guidelines, and more data). Doubts about financial viability, a perceived low likelihood of approvals of some medicines and other concerns prevented some company applications.

The desire for more data echoes previous German research [15, 18]. This desire emerged stronger than for similar research in other countries [4]. Pre-reclassification and post-reclassification research would enhance evidence-based decision-making and indicate possible improvements in the supply model. Three-years’ market exclusivity linked to informative pre-reclassification studies (like the US) [12] or post-reclassification surveillance (like Japan) [12] has been suggested [30], and would encourage the desired research. The longer market exclusivity period has been mooted before by industry, but this research indicates clear benefits to decision-making and understanding post-reclassification outcomes. Educating the committee in observational research could help.

The committee was mostly reported to work well and was largely considered evidence-based, although more evidence might help reduce some reliance on opinions. To achieve a benefit-risk consideration, the EU switch guide [24] (in which all four criteria concentrate on risk) could include more about benefit. Many committee members and industry would welcome flexibility with conditions to enable reclassifications, e.g. initial doctor diagnosis; flexibility has helped reclassification elsewhere [12]. A range of expertise on the committee probably aided decision-making, unlike Australian state representatives subject to political pressure [20], although community pharmacy expertise was lacking. While some committees considering reclassification have patient or consumer representatives [20], this concept received little support because of the scientific focus, as in other health services areas [31]. As in the US [21, 32], committee members could sometimes prepare better. Splitting off the veterinary aspects (as in Australia) [20], thereby reducing workload, and paying committee members might help.

The finding that some company applications and expert credibility could improve is similar to US research [21]. Advance sharing of the regulator’s evaluation (as in some other jurisdictions) could be trialled to help maximise the benefit of the company expert’s attendance at the meeting.

The post-committee process differs from elsewhere in Europe [22], delaying or rejecting reclassifications the committee recommends. Process timelines and publishing reasons for varying from the committee’s recommendation would help stakeholders. Streamlining, e.g. through a single agency involvement, or allowing a Ministry of Health delegate to approve the reclassification (as elsewhere) [20], would help address concerns.

Automatic loss of reimbursement discourages some reclassifications (as found elsewhere) [12]. Allowing continued reimbursement in some cases may be cost-effective for insurers and aid equitable access.

Support may aid good practice in pharmacy as indicated with the emergency contraceptive, but evidence is needed. Concerns from committee members and others about consumer behaviour may partly arise from the little evidence from Germany on non-prescription medicine usage [3]. Alternatively, it might reflect conservatism, similar to Australia where concerns about consumer behaviour particularly hindered reclassification [20].

The mixed views on access to doctors and thus the need for reclassification perhaps reflected the participant’s location, as such access varies considerably in Germany [33].

Other reclassification research found global influences important [12, 20, 30], unlike this research. This may reflect Germany’s large and important market.

### Strengths

The many interviews, ‘insider’ approach, transcription checking, and independent review are strengths. Ten committee members and a range of stakeholders provided a breadth of views and experiences. The ‘insider’ approach helps rapport with and efficiency for busy participants and the study [27]. The single interviewer allowed accumulated knowledge over the project.

### Limitations

Using a single ‘insider’ researcher could insert researcher biases. To counter this, the researcher remained aware of her biases, sought a range of perspectives, fact-checked with participants during reporting, checked diagrams with participants, coded and analysed the data systematically, and had independent review. The researcher attempted to reflect participants’ voices accurately with balance in analysis and reporting.

Interviews would likely differ with another interviewer, but participants were senior people, usually very involved with reclassification, and could modify their transcript. Greater descriptions of participants when quoted would help the reader, but could reveal participants.

## Conclusion

Reclassifying medicines can aid timely consumer access and health system efficiency. Although enablers to reclassification in Germany exist, such as the large non-prescription market, self-medication culture and significant pharmaceutical industry, barriers delay or prevent reclassifications that have happened in other countries. Barriers include committee conservatism, generic competition, insufficient evidence, and using conditions on supply such as initial doctor diagnosis only rarely. Recommendations to overcome barriers include collection of more data, application improvements, pharmacy support (including through under-graduate education) and evidence of pharmacy’s role in self-medication. For industry, strong immediate generic competition limits viability of reclassifications and research. Greater data collection, including post-reclassification, for the committee, government and society, could be incentivised by a longer market exclusivity. Committee and process improvements could also help.

## Data Availability

The data (transcripts or NVIVO analysis) is not available as it could identify the participants.

## Abbreviations

BfArM: Bundesinstitut für Arzneimittel und Medizinprodukte
EU: European Union
FDA: Food and Drug Administration
UK: United Kingdom
US: United States
